# Bract size affects resource availability and fruit set in a hummingbird‐pollinated plant with distyly polymorphism

**DOI:** 10.1111/plb.70079

**Published:** 2025-07-28

**Authors:** R. Trevizan, P. E. Oliveira, V. L. G. Brito, L. Oliveira, F. Telles

**Affiliations:** ^1^ Programa de Pós‐Graduação em Biologia Vegetal Universidade Estadual de Campinas Campinas Brazil; ^2^ Programa de Pós‐Graduação em Ecologia, Conservação e Manejo da Fauna Silvestre Universidade Federal de Minas Gerais Belo Horizonte Brazil; ^3^ Instituto de Biologia, Universidade Federal de Uberlândia, Campus Umuarama Uberlândia Minas Gerais Brazil; ^4^ Programa de Pós‐graduação em Ecologia, Conservação e Biodiversidade Instituto de Biologia, Universidade Federal de Uberlândia, Campus Umuarama Uberlândia Minas Gerais Brazil; ^5^ Instituto Nacional de Ciência e Tecnologia Polinização, INPol Jardim Botânico do Rio de Janeiro Rio de Janeiro Brazil

**Keywords:** floral polymorphism, floral reward, honest signalling, pollination

## Abstract

- Plants can use non‐floral signals to advertise the presence of resources to pollinators. The distylous *Psychotria poeppigiana* (Rubiaceae) has reddish bracts with small yellow flowers. Similar to other bracted plants with inconspicuous flowers, such bracts may signal the presence of nectar to pollinators.
- We investigated whether bracts act as honest signals in *P. poeppigiana* and how variation in bract traits affects floral reward and fructification. We asked: (1) Is there a relation between attractive traits (i.e. bracts and flowers) and the availability (quantity) and quality of the resource offered? (2) Do bract traits influence fructification rate? We hypothesized that bracts act as an honest signal to pollinators, being positively associated with nectar quantity and quality. If the signal is honest, we also expected that bracts with more resources could potentially attract more visits and result in a higher fruit set. We tested: (1) bract and flower trait differences between long‐styled (L‐styled) and short‐styled (S‐styled) morphs; (2) the relationship between bract (size, shape, asymmetry, and colour) and flower (length and diameter) traits and floral resource availability and quality; (3) bract trait effect on fruit set; and (4) whether bract size influences the total number of flowers and bract mortality.
- Larger bracts were positively associated with nectar volume, number of flowers, and increased bract mortality. In contrast, smaller bracts were linked to higher fruit set. Bract colour and asymmetry had no significant effect on resource production or fruit set. We additionally found differences between morphs: the S‐morph had larger bracts (10.37%), longer flowers (13.67%), and more flowers per bract (22%) than the L‐morph.
- We conclude that bracts in *P. poeppigiana* act as an honest signal to pollinators, as larger bracts produced more nectar. The higher fruit set in smaller bracts and increased mortality in larger ones suggest a potential division of reproductive roles, with S‐styled (larger bracts) flowers contributing to male reproductive function and L‐styled (smaller bracts) to female function.

## Graphical abstract

In *Psychotria poeppigiana*, larger bracts honestly signal greater nectar availability but have higher mortality, while smaller bracts produce more fruit, revealing a surprising trade‐off and hints of sexual role specialization in this distylous species.

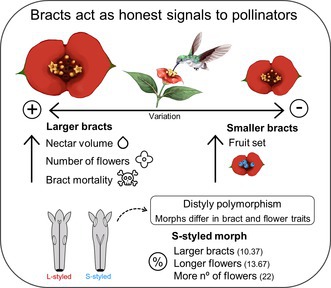

## INTRODUCTION

Flowers and pollinators have an offer–reward relationship, where animals visit flowers to obtain resources for their diet and/or reproduction (Kearns & Inouye 1997; Bronstein *et al*. 2006; van der Kooi *et al*. 2021). Plants, in return, obtain conspecific pollen deposited on stigmas, resulting in pollination and, later, fertilization of flowers (Sprengel 1793; Ollerton *et al*. 2011; Johnson & Schiestl 2016). The relationship between plants and pollinators is often viewed as a cooperative system that benefits both parties (Sun *et al*. 2018). However, such interaction requires a significant energy cost; thus, it is common for plants and animals to deceive each other (Bronstein 2001; Johnson & Schiestl 2016).

Plants attract animals to their flowers by advertising the presence of some resource that can involve single or several sensory signals, such as odour (e.g. Wright & Schiestl 2009; Schiestl & Dötterl 2012; Farré‐Armengol *et al*. 2013; Knauer & Schiestl 2014; Raguso *et al*. 2015), tactile (e.g. von Helversen & von Helversen 1999; Helversen *et al*. 2003; Simon *et al*. 2011), and/or visual (e.g. Medel *et al*. 2003; Brito *et al*. 2015; van der Kooi *et al*. 2019; Santana *et al*. 2022; Scaccabarozzi *et al*. 2022) signals. Although plants might produce floral signals indicating the presence of a reward, not all plants provide resources to their visitors (rewardless plants; Chittka & Raine 2006; Johnson & Schiestl 2016; Cardoso *et al*. 2023). For this reason, the signals displayed by plants can be considered honest or dishonest, with honest signals involving the true presence of a reward to their pollinators, such as nectar, pollen, oils, and others, while dishonest signals involving the deception of pollinators, with rewardless flowers (Bolstad *et al*. 2010; Johnson & Schiestl 2016; van der Kooi *et al*. 2023).

In addition to detecting the presence of a signal associated with the presence of a reward, traits like signal size, shape, symmetry, display, and colour variation could also relate to the quantity and/or quality of resources being offered, which are ultimately perceived and learned by visitors (Gegear & Laverty 2001; Giurfa 2001; Makino & Sakai 2007; Dyer & Murphy 2009; Knauer & Schiestl 2014; Ne'eman & Ne'eman 2017). For example, it has been shown that a large corolla is positively correlated with the amount of reward (pollen allocation and nectar), increasing pollinators' visitation (Stanton & Preston 1988). Also, studies have demonstrated that signal size, such as a larger floral display, is associated with a greater number of pollinator visits, enhancing the chances of successful pollination (Klinkhamer *et al*. 1989; Harder & Johnson 2005; Makino & Sakai 2007; Brys & Jacquemyn 2010; Hernández‐Villa *et al*. 2020; Cunha & Aizen 2023). Floral symmetry and colour have also been shown to influence pollinator preference, as they can reflect higher nectar production or quality, potentially increasing pollinator visitation (Møller 1995; Varga & Soulsbury 2017).

The plant's signalling does not always appear in the flower parts, such as petals and stamens (Brito *et al*. 2015), but can also occur in nonfloral structures, such as bracts (Armbruster *et al*. 2005; Pérez‐Barrales *et al*. 2013; Bergamo *et al*. 2019), specialized leaves often found subtending or surrounding flowers. Bracts can act in pollinator attraction, particularly when the floral unit is inconspicuous over long distances, thereby enhancing the overall attractiveness of the plant (Herrera 1997; Borges *et al*. 2003; Armbruster 2023). Bracts can also serve as a sensorial defence mechanism when flowers deliberately avoid their larcenists by sensory exclusion through the colour of the bracts (Armbruster *et al*. 2005; Keasar *et al*. 2006; Pélabon *et al*. 2012; Bergamo *et al*. 2019). It has been shown that bracts can provide less contrast for bees and thus reduce the chances of less effective floral visitors or bee nectar robbers (i.e. bee avoidance hypothesis; Lunau *et al*. 2011; Bergamo *et al*. 2019). In other cases, the presence of bracts effectively disguises the floral structure and significantly minimizes floral herbivory (Klooster *et al*. 2009; Lev‐Yadun 2016), acting as a defence mechanism against antagonists, and thereby enhancing the plant's reproductive fitness (Armbruster *et al*. 2005; Klooster *et al*. 2009).

**Fig. 1:**
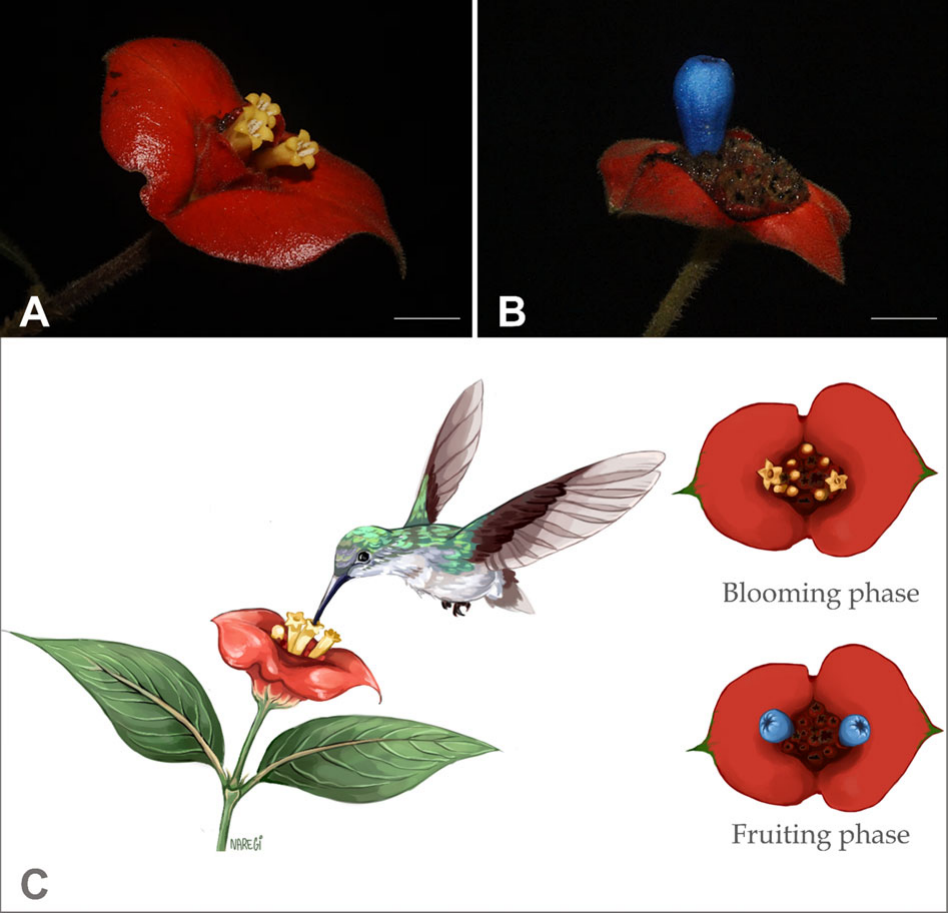
(A) The inflorescence of *Psychotria poeppigiana* (Rubiaceae) consists of a bright reddish bract with yellow flowers immersed in the center of the structure during the flowering stage. (B) Bract with a purple fruit during the fruiting phase. (C) Representation of the studied population system, with flowers organized in terminal and capitate inflorescences emitted between a pair of leaves. The main pollinators considered are hummingbirds, represented here by *Thalurania furcata* (Trochilidae). Ilustration by Giovana Narezi.

The inflorescences of *Psychotria poeppigiana* Müll. Arg. (Rubiaceae) are characterized by being surrounded by showy reddish bracts that exhibit prolonged longevity, emerging on the plant from the pre‐flowering stages until the end of the fruit dispersal period (Fig. 1A, B). Each inflorescence presents small yellow and tubular flowers immersed in the center of the bract, which, at a certain distance, could make them difficult to detect by pollinators, mainly hummingbirds (Fig. 1C; Coelho & Barbosa 2004; Valois Cuesta *et al*. 2009). Similar to other conspicuous bract plant species with inconspicuous flowers that hummingbirds visit (i.e. Bergamo *et al*. 2019), the bracts of *P. poeppigiana* may be operating as a reward signal for their floral visitors. Nevertheless, the honesty of the signal remains unknown. In addition, *P. poeppigiana* exhibits variation in bract size, shape, and symmetry within and among individuals. Also, the population presents distinct floral morphs, that is, distyly polymorphism, in which two morphs co‐occur in populations: a long‐styled morph (L‐styled) and a short‐styled morph (S‐styled), corresponding reciprocally to each other in the height of their sexual structures. Although the relationship between bract traits and floral morphs remains unexplored, studies have shown differences in pollinator attraction traits and reproductive response between morphs of distylous species (Ornelas *et al*. 2004; Cawoy *et al*. 2006; Wang *et al*. 2022). For instance, in *Tirpitzia sinensis* (Linaceae), the L‐morph produces a larger volume of nectar but with a lower sucrose‐to‐hexose ratio than the S‐morph. This difference may extend pollinator foraging time and enhance pollination efficiency (Wang *et al*. 2022). Similarly, in *Palicourea padifolia* (Rubiaceae), floral morphs also differ in nectar volume and sugar composition, although these differences did not lead to variation in reproductive success (Ornelas *et al*. 2004). Such trait variation highlights the potential role of floral rewards in modulating pollinator attractiveness, influencing the quantity and quality of the reward offered, and ultimately affecting fruit set.

Based on this context, this study aimed to investigate the significance of bract traits on reproductive success by analysing how variation in such structure affects floral reward and reproductive success. Additionally, we explored whether floral traits contribute to resource availability and quality, and explored differences between morphs in bract and floral traits. We specifically addressed the following questions: (1) Is there a relationship between attractive traits (i.e. bracts and flowers) and the quantity and quality of the reward offered? (2) Do bract traits, such as size, shape, asymmetry, and colour, influence the rate of fructification? We hypothesized that attractive bract traits, such as size, shape, asymmetry, and colour (i.e. chromatic contrast with the background), act as an honest signal to pollinators. Specifically, we expected larger, more conspicuous, and more symmetrical bracts to be positively associated with resource quantity/quality. If the bract signal is honest, we also expected that bracts associated with greater floral resources could enhance pollination success and, under efficient pollination, translate into higher fruit production compared to bracts with fewer or no resources.

## MATERIAL AND METHODS

### Studied site and system model

The study was conducted between October 2020 and January 2021 in a gallery forest in the natural reserve of the Clube Caça e Pesca Itororó (127 ha; 18°55′S; 48°17′W), Uberlândia, Minas Gerais, Brazil. The area is dominated by Cerrado, the Brazilian savanna.

*Psychotria poeppigiana* is a subshrub commonly found in seasonally flooded forest groves (Coelho & Barbosa 2004; Valois Cuesta *et al*. 2009). The flowering period is from June to January, and fruiting from October to March (Coelho & Barbosa 2004). Flowers are organized in capitate, terminal inflorescence (the bract), which emerges between a pair of leaves (Fig. 1; Coelho & Barbosa 2004). Flower number varies among the bracts, ranging from five to 34 flowers per bract (mean ± SD: 12.71 ± 5.27). Each inflorescence belonged to a branch individually marked at the beginning of the study. The inflorescences are surrounded by reddish fused bracts that remain turgid from the beginning of flowering until the end of the fruiting period. However, there is a high rate of bract mortality (i.e. spontaneous fall of bracts) during both phases, leading to the premature loss of flowers and developing fruits (R.T. and F.T. pers. obs.). The inflorescences contain odourless, yellow tubular flowers, and offer nectar as a floral reward for pollinators, which are predominantly hummingbirds (Fig. 1; R.T. pers. obs.; Coelho & Barbosa 2004). *Psychotria poeppigiana* exhibits distyly, with individuals of two distinct floral morphs: A long‐styled morph (L‐morph or pin) with stigma above the anthers, and a short‐styled morph (S‐morph or thrum) with anthers above the stigma (Cardoso *et al*. 2018). The reciprocal positioning at the height of the anthers and stigmas allows for efficient pollen transfer between distinct floral morphs, promoting intermorph cross‐pollination in the populations (Darwin 1877; Keller *et al*. 2014; Trevizan *et al*. 2021). Despite being characterized as xenogamous with self‐ and intramorphic incompatibility system (Coelho & Barbosa 2004), *P. poeppigiana* also presents vegetative growth (i.e. clonal propagation), forming clusters of the same individual within the population.

To answer the research questions, we initially chose 25 individuals (11 L‐morph and 14 S‐morph) in which we studied the morphological bract and flower traits, nectar production, and finally, reproductive success measured in terms of fruit set. Given the clonal growth habit of the species and the inherent difficulty in delineating genet boundaries, we defined each spatially distinct cluster of the same floral morph as a single individual. The minimum distance between individuals of different morphs was ca. 1 m, while individuals of the same morph were separated by distances >1 m. Throughout the study, some individuals and inflorescences were lost due to bract mortality, so the response variables could not be measured in all individuals. Because our interest was focused on evaluating the effect of the bract traits and not on the effect of the individual, the final analyses were carried out only with the individuals for which all data could be collected. The sample size used in the study is provided in Table S1.

### Bract traits: Shape, size and asymmetry

To investigate variations in size, shape, and asymmetry in bracts—traits that could potentially function as signals to pollinators—we standardized photographs of bracts from selected individuals (Camera Canon Ti3 Eos; Lens Ef‐s 18‐55 mm), positioning bracts on graph paper perpendicular to the camera lens focal plane. To assess 2D morphological variation, we utilized Geometric Morphometric methods (GMM) (Bookstein 1991; Zelditch *et al*. 2012). The images were prepared and organized using the *TPSUtil* program (v. 1.80; Rohlf 2015), and landmarks and semi‐landmarks were digitized using the *tpsDig* program (v. 2.31; Rohlf & Slice 1990; Zelditch *et al*. 2012; Rohlf 2015). During the digitalization process, all images were thoroughly scaled. We defined the landmarks and semi‐landmarks following Zelditch *et al*. (2012), considering points 1–4 as landmarks, represented by the left and right bract extremities (landmarks 1 and 3) and the bract structure entrances (landmarks 2 and 4). Semi‐landmarks were distributed contouring the bract's edge surface (5–16; Zelditch *et al*. 2012; Gunz & Mitteroecker 2013). The subsequent geometric morphometric analysis was performed using the *geomorph* package in software R (v. 4.0.5; Adams *et al*. 2023).

We defined the coordinates of landmarks and semi‐landmarks using the *define.sliders* function. After this, we performed Generalized Procrustes Analysis (GPA) to obtain shape variables by removing the non‐shape components (i.e. size, location and orientation) using the *gpagen* function (Zelditch *et al*. 2012; Savriama 2018). To visualize the similarities and differences among bracts, we performed principal components analysis (PCA) on Procrustes shape coordinates using the *gm.prcomp* function. Relative Warp (RW) values were obtained from the PCA. These values represent the transformed shapes of the bracts along the variability axes of the morphospace, quantifying their variation in each individual. We extracted the bract size information through the centroid size (cs) for each bract sample. The cs is calculated as the square root of the sum of all landmarks' squared distances from their centroid, being considered a measure of size. To quantify components of shape variation, we evaluated the directional asymmetry (i.e. variation among sides of each bract sample) using the *bilat.symmetry* function (Klingenberg *et al*. 2002; Savriama 2018; Adams *et al*. 2023).

### Bract colour

To evaluate bract colour and its potential signalling function, as well as its influence on reproductive success, we used spectrophotometry and the tetrahedron colour space modelling to calculate the colour contrast of bracts against the background (Stoddard & Prum 2008). We measured the spectral reflectance of the bracts (n = 68) using a portable spectrophotometer (Jaz; Ocean Optics, Dunedin, FL, USA) coupled with an optic fibre (R400‐7‐UV–VIS Jaz; Ocean Optics) linked to a holder that allowed us to take the measurements at an incidence angle of 45°. We used a pellet of barium sulfate as the white standard and covered the spectrophotometer entrance as a black standard for calibration. With the reflectance curves of each bract, we built a tetrahedron colour space considering a standard ideal illumination and a standard function of green leaves, both from the package ‘*pavo*’ (Maia *et al*. 2019). Since the main pollinators of *P. poeppigiana* are hummingbirds, we used the relative sensitivity of the avian UV system (Vorobyev *et al*. 1998) available in the same package. In the tetrahedron colour space, colours are represented as dots in a three‐dimensional space, and colour differences are measured as the Euclidean distance between dots. Therefore, we used the distance between the dot representing each bract and the center of the tetrahedron (r.vector) as a measure of colour contrast against the foliage background.

### Flower traits

In the same selected individuals, the number of flowers per inflorescence was counted. To measure flower traits, lateral photographs of between four and seven flowers per inflorescence were taken using graph paper as a background (Camera Canon Ti3 Eos; Lens Ef‐s 18–55 mm). We recorded the measurements using the *ImageJ* software (Schneider *et al*. 2012). Flower length and diameter, both considered attractive floral traits, were measured. Flower length was defined as the distance from the corolla base to the petal opening. Flower diameter (corolla opening) was measured using an electronic calliper (316119 MTX).

### Availability (quantity) and quality of floral resource

In the same flowers used for the morphometric analysis, the amount and concentration of nectar they produced per day were estimated (Table S1). We used nectar volume (μL) and total sugar (energy) content as metrics to estimate floral resource availability (quantity) and quality. To determine the amount of nectar that flowers can produce each day, we measured cumulative nectar production in pre‐anthesis bagged flowers (to avoid floral visitors). Nectar volume was collected in capillary tubes (20 μL), and the sugar concentration (%) was measured using a hand refractometer (Brix 0%–90% RZ; Galetto & Bernardello 2005). We used the equation *y* = 0.00226 + (0.00937×) + (0.0000585 ×2) to calculate sugar per μL (unit of volume) of nectar produced by each flower, where ‘*y*’ is mg of solutes per μL and ‘*x’* is the refractometer value reading (i.e. sugar concentration; Galetto & Bernardello 2005). The total sugar (energy) content per flower was calculated by multiplying ‘*y*’ by the total volume.

### Reproductive success mediated by bracts

Finally, for a subset of the marked inflorescences with known bract traits, fruit production was measured (Table S1). To assess reproductive success, we counted the number of flowers and flower scars on bracts, and recorded the number of developed fruits. We monitored the plants for 2 months, which corresponds to the fruiting period of the population, allowing us to evaluate the effectiveness of pollination. We also quantified the number of individualized bracts that fell (i.e. bract mortality) during the study period.

### Statistical analyses

We first checked the occurrence of multicollinearity among the predictor variables (bract size, shape, asymmetry, colour, flower length, and diameter) by calculating their Variance Inflation Factors (VIFs) using the *vifstep* function in the *usdm* R‐package (Naimi *et al*. 2014). We excluded the variables with VIF values >3, as suggested by Zuur *et al*. (2010). Shape and asymmetry were found to be collinear, and shape was randomly excluded from the analyses. The remaining five variables are listed in Table S2. We also assessed pairwise correlations between these predictor variables using the *chart.Correlation* function of the *PerformanceAnalytics* R‐package. The resulting correlation matrix is presented in Fig. S1.

We explored differences between morphs in terms of bract and floral traits, which were treated as response variables in our analyses. Specifically, we considered bract size, asymmetry, and colour (bract traits), along with flower length, flower diameter, and number of flowers per bract (floral traits), to assess distinctions between L‐ and S‐styled floral morphs. To accomplish this, we constructed separate models for each response variable, using floral morph as the predictor variable and individual as a random effect. We applied a generalized linear mixed model (GLMM) with *Gamma* distribution and *log* link function (continuous/positive data), except for the number of flowers response variable, where we used *binomial negative 2* distribution and *log* link function (count data) (Dunn & Smyth 2018).

To analyse whether attractive morphological traits (i.e. bract size, bract asymmetry, bract colour, flower length, and flower diameter) were related to floral resource availability and quality (i.e. volume of nectar in μL and energy content of nectar in mg), we applied two GLMM with *Gaussian* distribution and *identity* link function (continuous/negative data) (Dunn & Smyth 2018). We considered attractive morphological traits and morph type as predictor variables, while branch nested within individual was the random effect. The response variables were volume of nectar in μL and energy content of nectar in mg, this last was log‐transformed to improve the model fit. We used the added zero‐inflation parameter in both models to account for the high number of zeros (i.e. flowers that did not produce nectar). Because bract colour had fewer observations compared to other predictor variables (n = 339), we performed a subset analysis to specifically evaluate the effect of bract colour on nectar availability and quality. We used the same model structure, selection, and assessment procedures as described previously.

To investigate whether reproductive success (i.e. fruit set) was affected by bract traits (i.e. size, asymmetry and colour), we applied a GLMM with *binomial* distribution and *logit link* function. We used bract traits and morph as predictor variables. To create our proportional response variable, we took the number of unformed fruits (i.e. number of failures) and the number of formed fruits (i.e. number of successes) and combined these two variables through the *cbind* function in R‐base package (Crawley 2013). We considered individual as random effect. Due to the small number of observations (n = 57 observations), we performed a subsetting to evaluate the effect of the nectar availability and nectar quality per bract on fruit set proportion. We used a GLMM with mean volume of nectar, mean energy content of nectar per bract, and morph type as predictor variables, and individual as the random effect. We applied the same procedures presented in the previous model for model selection and assessment.

Subsequently, we investigated the influence of bract size on the total number of flowers per bract. We applied a GLMM with *binomial negative 2* distribution and *log link* function. We used bract size as the predictor variable and individual as the random effect. We also investigated the influence of bract size on bract mortality. We utilized a GLMM with a *binomial* distribution and a *logit link* function. Bract size was a predictor variable, and the individual was the random effect. We calculated the effect size of the model using the *r2_tjur* function from the *performance* R package (Lüdecke *et al*. 2021).

We used the *glmmTMB* R package to fit all models (Brooks *et al*. 2017). For each model, we checked adjustment using the QQ plot of residuals and the plot of predicted *vs*. residual values, by simulating residuals 250 times in the *DHARMa* R package (v. 0.4.5; Hartig & Lohse 2022). All diagnostic plots are provided in Fig. S2. To test the significance of the models, we applied a type II Wald Chi‐square test using the *car* R package (v. 3.1.0; Fox *et al*. 2018). After detecting significance, we calculated both marginal and conditional *R* ^2^ using the *r2_nakagawa* function from the *performance* R package (v. 0.10.2; Nakagawa *et al*. 2017; Lüdecke *et al*. 2021). To report and plot results, we used model‐adjusted values that were back‐transformed using the *ggpredict* function from the *ggeffects* R package (v. 1.1.4; Lüdecke 2018). All analyses were performed in the software R (R Core Team 2023).

## RESULTS

**Fig. 2:**
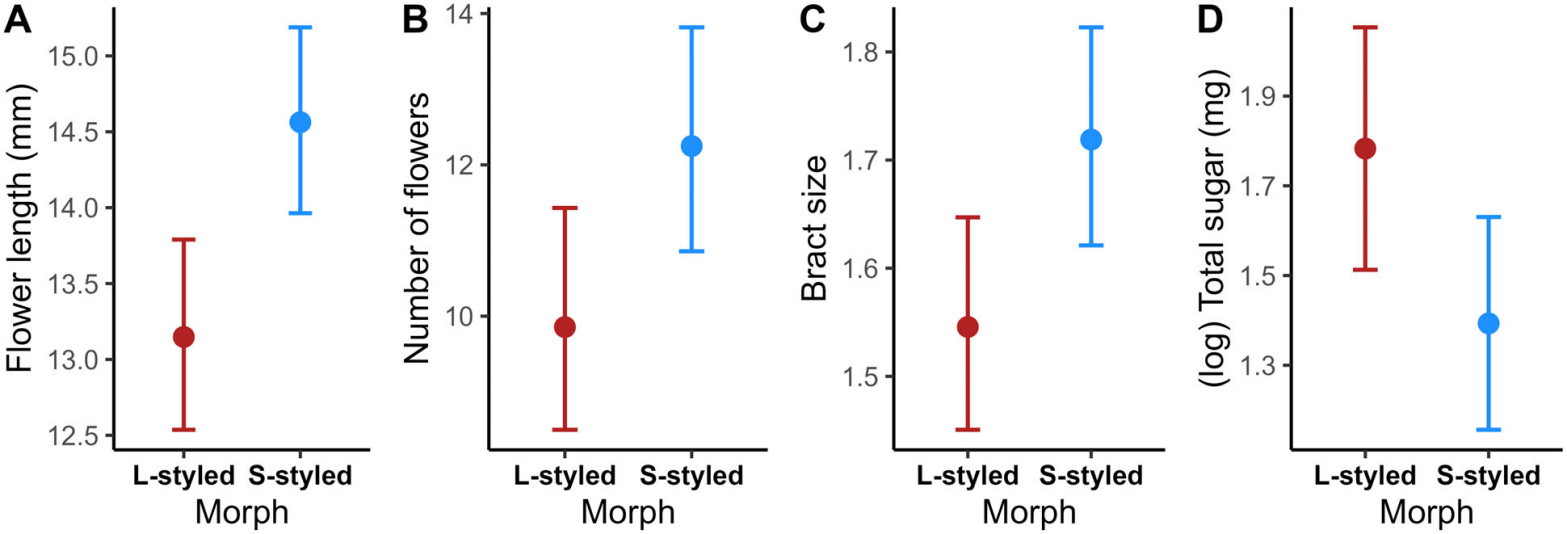
Differences between floral morphs (L‐styled and S‐styled) and (A) flower (corolla) length (mm), (B) number of flowers, (C) bract size (centroid size—cs), and (D) nectar energy content (total sugar in milligrams). Red dots (L‐styled) and blue dots (S‐styled) and error bars show back‐transformed marginal (model‐adjusted) means ± SE.

We confirmed the variation of bract and flower traits between morphs. The flower length of the S‐styled morph was 13.67% higher than that of the L‐styled morph, with *R* ^2^_marginal_ of 0.123 and a *R* ^2^_conditional_ of 0.413 (*χ* ^2^ = 9.938, df = 1, *P* < 0.001; Fig. 2A, Table S3). The number of flowers per bract also differed between morphs. The S‐styled morph was 22% higher in total flower number than the L‐styled morph, with an *R* ^2^_marginal_ of 0.040 and a *R* ^2^_conditional_ of 0.277 (*χ* ^2^ = 7.806, df = 1, *P* = 0.005; Fig. 2B, Table S3). Bract size of S‐styled morph was 10.37% higher than that of the L‐styled morph, with a *R* ^2^_marginal_ of 0.067 and an *R* ^2^_conditional_ of 0.353 (*χ* ^2^ = 12.21, df = 1, *P* < 0.001; Fig. 2C, Table S3). There was no significant difference between L‐ and S‐styled morphs for bract asymmetry, bract colour, or flower diameter (Table S3).

**Fig. 3:**
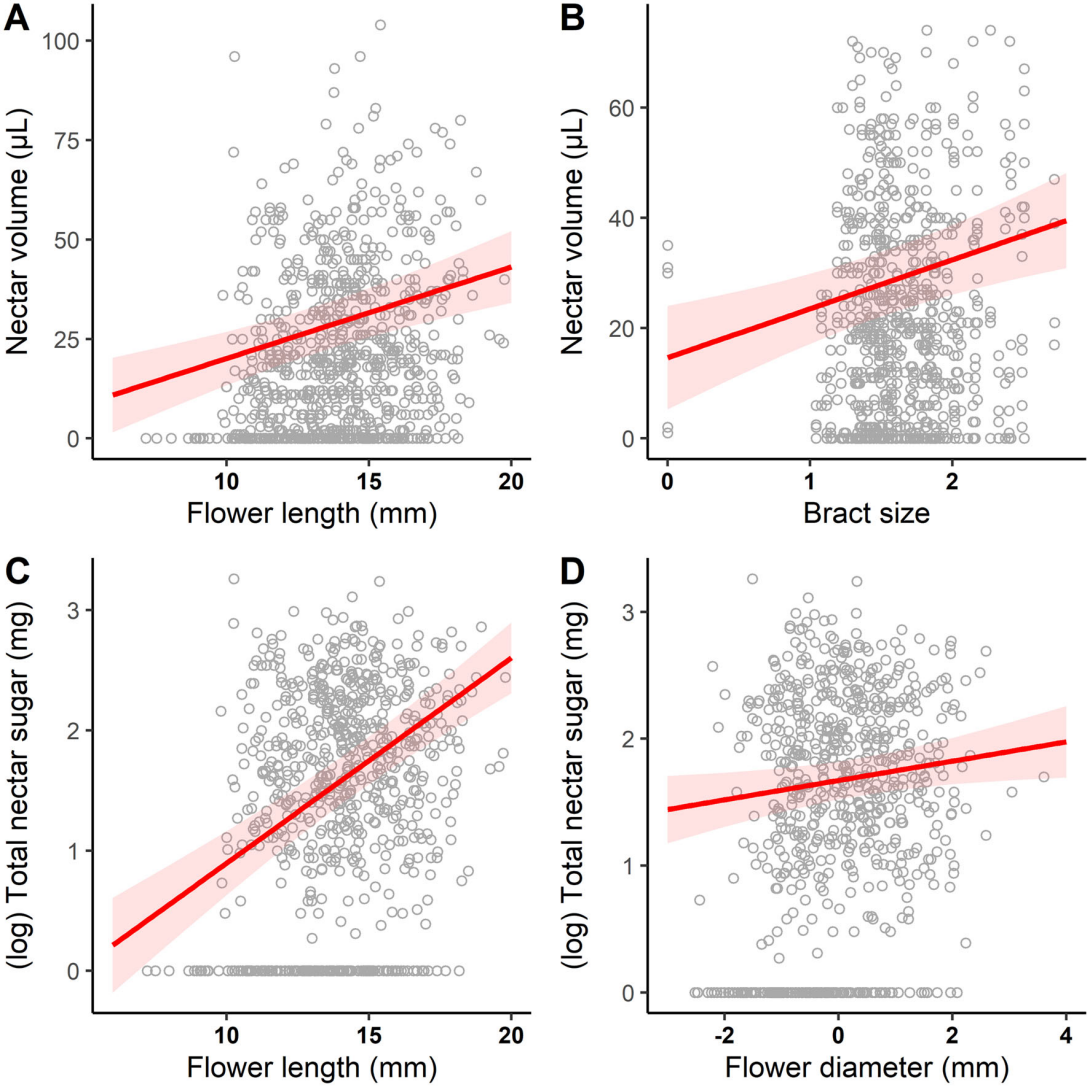
Relationship between bract and flower morphological traits on the availability (quantity) and floral resource quality. Effect of predictor variables (A) flower length and (B) bract size on nectar volume (response variable), and (C) flower length and (D) flower diameter on total nectar sugar (response variable). The red line indicates the plotted model. The red bands indicate the 95% confidence interval. The points on the graphs correspond to the raw data.

Regarding floral resource availability, we found a significant effect of bract and flower traits on nectar volume, with *R* ^2^_marginal_ of 0.22 and *R* ^2^_conditional_ of 0.87. Flower length had a positive effect, explaining 10.42% of the variance (*χ* ^2^ = 19.784, df = 1, *P* < 0.001; Fig. 3A, Table S4). Bract size had a positive effect, explaining 2.94% of the variance (*χ* ^2^ = 12.77, df = 1, *P* < 0.001; Fig. 3B, Table S4). The remaining predictor variables did not significantly affect nectar volume (Table S4). For floral resource quality, we found a significant effect only of floral traits on nectar energy content, with an *R* ^2^_marginal_ of 0.126 and an *R* ^2^_conditional_ of 0.443. Flower length had a positive effect, explaining 13% of the variance (*χ* ^2^ = 68.773, df = 1, *P* < 0.001; Fig. 3C, Table S4). Although relatively low, flower diameter also had a positive effect, explaining 0.8% of the variance (*R* ^2^_marginal_ 0.008; *χ* ^2^ = 5.136, df = 1, *P* = 0.023; Fig. 3D, Table S4). There was also a significant difference between morphs, with L‐styled morphs having, on average, 4.97% more nectar energy than S‐styled morphs (*χ* ^2^ = 4.390, df = 1, *P* = 0.036; Fig. 2D, Table S4).

**Fig. 4:**
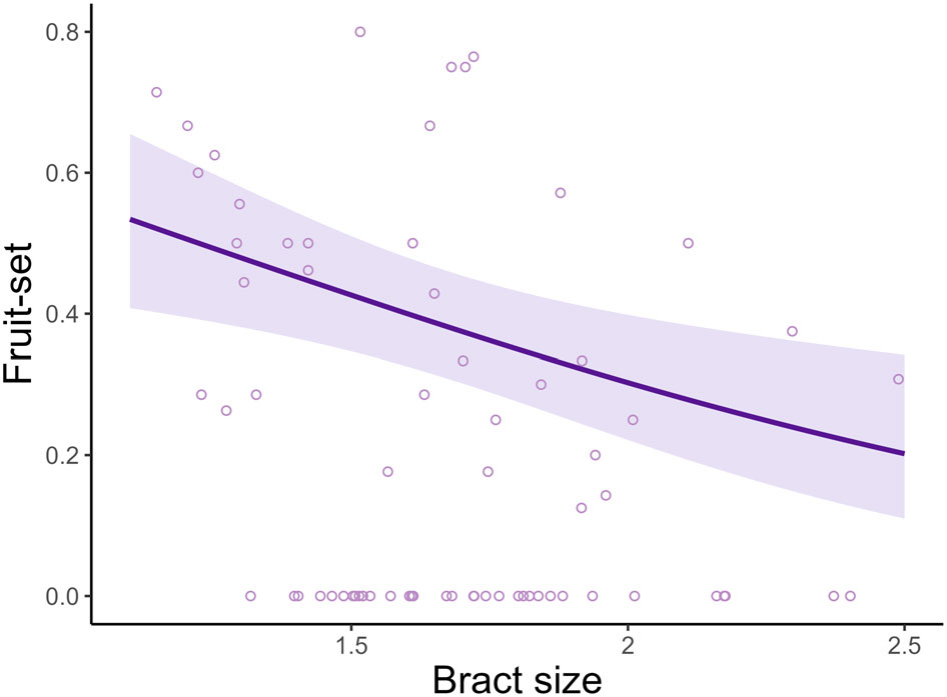
The negative effect of bract size (predictor variable) on fruit‐set (response variable). The line indicates the plotted model. The purple bands indicate 95% confidence interval. The points on the graph correspond to the raw data.

Regarding reproductive success affected by bract traits (i.e. bract size, asymmetry, and colour), only bract size had a significant effect, presenting a negative effect on fruit‐set proportion, with an *R* ^2^_marginal_ of 0.242 and an *R* ^2^_conditional_ of 0.286 (*χ* ^2^ = 8.281, df = 1, *P* = 0.004; Fig. 4, Table S5). Bract colour, asymmetry, and floral morph did not significantly affect fruit‐set (Table S5). The resource availability and quality of nectar per bract did not have a significant effect on fruit‐set (Table S5).

**Fig. 5:**
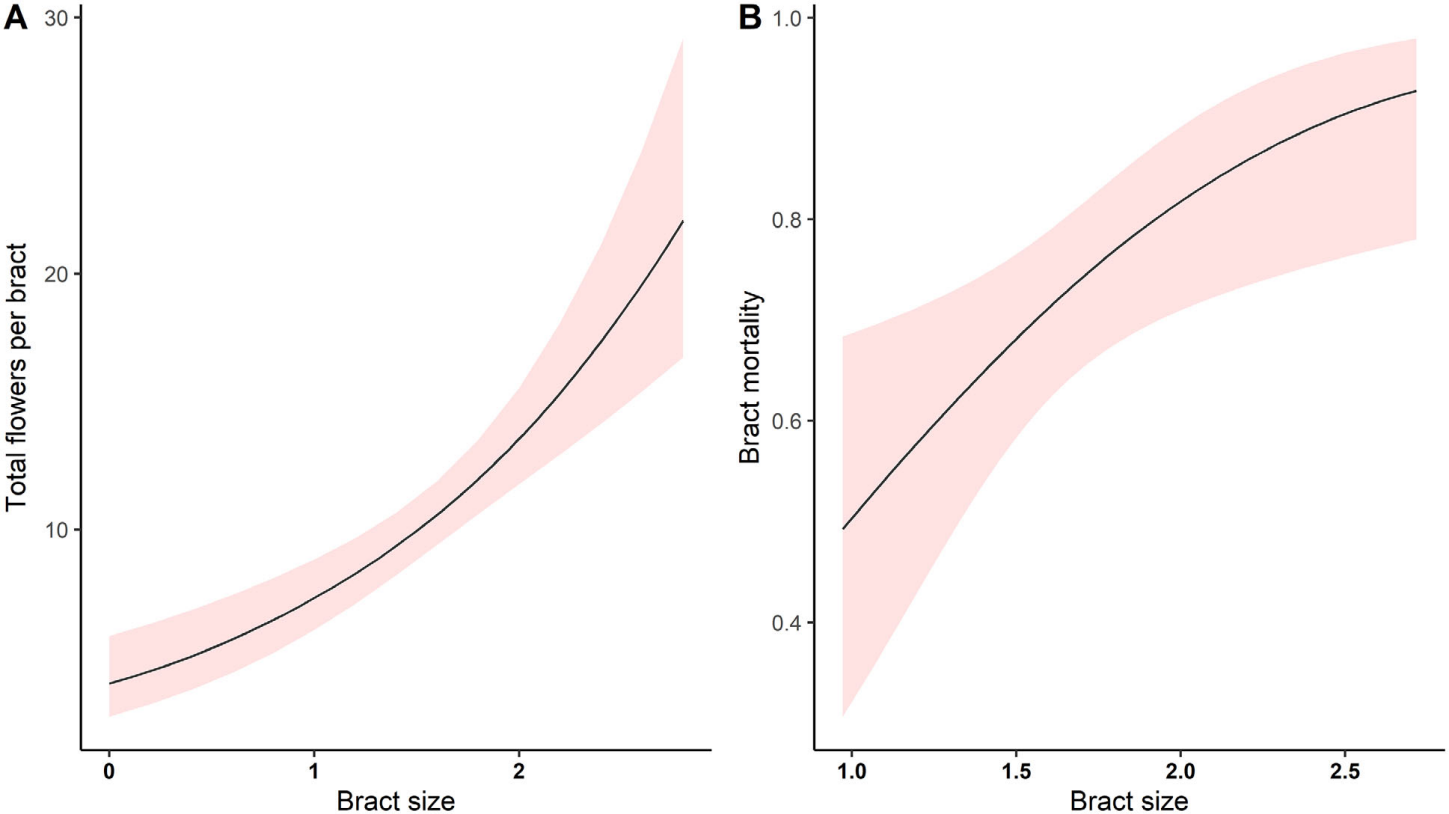
(A) Positive effect of bract size (predictor variable) on number of flowers per bract (response variable) and (B) positive effect of bract size (predictor variable) on bract mortality (response variable). The line indicates the plotted model. Red bands indicate 95% confidence interval.

Bract size impacted the total number of flowers per bract and the bract mortality rate. Bract size presented a positive effect on number of flowers, with the number of flowers increasing with the size of bracts (*R* ^2^_marginal_ of 0.164 and an *R* ^2^_conditional_ of 0.375; *χ* ^2^ = 29.405, df = 1, *P* < 0.001; Fig. 5A, Table S5). Bract size also had a positive effect on bract mortality, with bract mortality increasing with the size of bracts (*χ* ^2^ = 7.046, df = 1, *P* = 0.007; Fig. 5B, Table S5), explaining 8.7% of the model's effect size (*R* ^2^__tjur_ = 0.087).

## DISCUSSION

In this study, we investigated the role of long‐lasting bracts as honest signals to pollinators, and how variation in bract traits related to floral reward and reproductive success in the distylous *P. poeppigiana*. We found differences between morphs, with S‐morphs having larger bracts and flowers, and producing more flowers per bract. Larger bracts and flowers produced greater volumes of nectar. Additionally, larger flowers (in both length and diameter) were associated with higher nectar energy content. L‐morph flowers exhibited higher nectar energy content than S‐morph flowers. Regarding the influence of bract traits on fruit production, we found that smaller bracts were associated with a higher fruit set. Additionally, our results revealed a positive relation between bract size and the total number of flowers. Interestingly, larger bracts also had higher mortality rates. The observed variation in bract colour and asymmetry was not significantly associated with morph type and did not explain differences in nectar quality, nectar volume or fruit set, suggesting that these traits vary independently across the population.

We predicted that bract traits would serve as the honest signals to pollinators, indicating that a larger signal of this non‐floral component could be linked to a higher quantity and quality of the provided resource. This was supported by observations in *Dalechampia ipomoeifolia* (Euphorbiaceae), where larger bract size correlated with a greater reward amount, serving as an honest signal to pollinators (Armbruster *et al*. 2005). We observed a similar pattern of higher nectar volume in larger bracts of *P. poeppigiana*. We also found a positive relation between flower length and nectar volume. Altogether, these trends indicate that bracts act as honest and reliable signal to hummingbirds in terms of the presence of nectar volume. Moreover, larger bracts exhibited a higher flower number (Fig. 5A), possibly having an even greater amount of floral resource.

Nevertheless, regarding nectar quality, L‐flowers had the highest levels of nectar sugar despite having significantly smaller bracts. Studies have pointed out differences between distylous morphs in traits relevant to the pollination process (Ornelas *et al*. 2004; Cawoy *et al*. 2006; Ferrero *et al*. 2017; Wang *et al*. 2022). For instance, S‐morph flowers have been shown to secrete a higher proportion of sucrose compared to L‐morph flowers in *Tirpitzia sinensis* (Linaceae), which was associated with S‐morph increased attractiveness to pollinators and thus visit rates (Wang *et al*. 2022). In our case, the L‐morph has smaller bracts. Although bract traits were not significantly related to nectar sugar content, having a higher sugar concentration in flowers may help maintain pollinator attraction and promote the disassortative (intermorph) pollination expected in the distyly polymorphism.

We found a negative relationship between bract size and fruit set, contradicting our initial hypothesis. We had expected that, if bracts were honest signals of resource availability and/or quality, larger bracts would attract more pollinators and lead to greater reproductive success compared to smaller bracts. Previous research in *Dalechampia ipomoeifolia*, for instance, found that the presence of larger bracts resulted in higher visitation rates and greater amounts of pollen on stigmas (Armbruster *et al*. 2005). However, as highlighted by Thomson (2025), increased visitation does not necessarily translate into a higher fruit set. Particularly in distylous species, successful fertilization depends on efficient intermorph pollen transfer (Keller *et al*. 2014). Therefore, the effect of bract traits on reproductive success likely involves additional mechanisms beyond pollinator frequency. Considering evidence that larger floral displays can enhance male fitness in plants by increasing pollen export (Cunha & Aizen 2023), it is plausible that larger bracts may contribute more to male reproductive success, while smaller bracts may optimize conditions for fruit and seed production (female fitness).

We also observed differences between floral morphs, notably with the S‐styled displaying larger bracts, larger flowers, and a greater number of flowers per bract. This opens new avenues for investigating the reproductive role of floral morphs, suggesting that the S‐morph may primarily serve a male function, facilitating pollen donation, while the smaller bracts, represented by the L‐styled morph, likely fulfil a female role, receiving pollen for seed formation. The gender specialization between floral morphs has been observed within distylous populations of other species, with the S‐morph usually specialized in exporting and the L‐morph in receiving pollen (Nicholls 1986; Ornelas *et al*. 2004; Watanabe *et al*. 2014). Further research into pollen traits, including pollen removal and deposition, will enhance our understanding of the sexual functions between morphs within a population and elucidate the distinct roles of bracts in the reproductive process.

Furthermore, we showed a positive relation between the bract size and the rate of bract mortality, indicating that larger bracts are linked to a higher incidence of bract mortality, a common event in the studied population (71.6%). The long‐lasting bracts of *P. poeppigiana* emerge during the pre‐flowering stage and persist until the conclusion of the fruit dispersal period. Maintaining attractive traits, in addition to producing nectar, requires additional energy costs to the plant (Ashman & Schoen 1996; Ashman 2004; Pyke & Ren 2023) and may potentially compete with other essential plant functions in the future, such as fruit development and seed production (Ashman & Schoen 1996; Pyke & Ren 2023). The high resource cost of maintenance may create a trade‐off, limiting or diminishing subsequent reproduction in larger bracts, leading to higher mortality. Consistent with this, larger bracts produced more flowers and nectar. This suggests again that larger bracts allocate more resources and may function as donors of pollen grains and nectar, falling after pollen transfer (i.e., bracts mortality). In contrast, smaller bracts, which present fewer flowers, may be directing their energy to fruit production.

We confirmed that bracts in *P. poeppigiana* serve as honest signals to pollinators, indicating the availability of floral reward. Our results present a new perspective on the differential roles in reproductive function of the morphs, with S‐styled morphs potentially serving a male function (larger bracts) and L‐styled morphs (smaller bracts) playing a female role within the population.

## AUTHOR CONTRIBUTIONS

RT and FT planned and designed the research. Data collection was done by RT, FT, and LO Analysis, figures, and writing of the first manuscript draft were done by RT, PEO, VLGB, and FT All authors read, provided suggestions, and approved the final version of the manuscript.

## CONFLICT OF INTEREST STATEMENT

The authors declare no conflicts of interest.

## Supporting information


**Table S1.** Number of plant individuals sampled, and total sample sizes of response variables used on models.
**Table S2.** VIFs of the remaining variables. Variables have been selected through ‘*stepwise*’ selection based on Variance Inflation Factors (VIF <3) to avoid multicollinearity among the predictor variables.
**Table S3.** Details of models concerning the differences between morphs in bract and floral traits, including the model parameters and their respective results.
**Table S4.** Details of models concerning the effects of attractive morphological traits on floral resource availability (nectar volume), and nectar quality (nectar sugar), including the model parameters and their respective results.
**Table S5.** Details of models concerning the effects of bract traits and resource on reproductive success, and the effect of bract size on the numbers of flowers and bract mortality, including the model parameters and their respective results.
**Figure S1.** Pairwise correlation coefficients among the continuous predictor variables (bract size, shape, asymmetry, colour, flower length, and diameter).
**Figure S2.** Quantile‐quantile (QQ) plots of residuals from the models.
