# Supplementary material for: Bract size affects resource availability and fruit set in a hummingbird‐pollinated plant with distyly polymorphism
Source: Plant Biol (Stuttg). 2025 Jul 28;27(7):1478–87. doi: 10.1111/plb.70079 (PMC12631528; doi:10.1111/plb.70079)

**SUPPORTING INFORMATION**

**Table S1.** Number of plant individuals sampled, and total sample sizes of response variables used on models.

| Response variable | Number of plant individuals | | | | Total number of flowers/bracts measured | | |
| --- | --- | --- | --- | --- | --- | --- | --- |
|  | L-styled | S-styled | Total | L-styled | | S-styled | Total |
| Bract shape | 11 | 14 | 25 | 103 | | 131 | 234 |
| Bract size | 11 | 14 | 25 | 103 | | 131 | 234 |
| Bract asymmetry | 11 | 14 | 25 | 103 | | 131 | 234 |
| Flower length | 11 | 14 | 25 | 320 | | 433 | 753 |
| Flower diameter | 11 | 14 | 25 | 320 | | 433 | 753 |
| Bract color | 8 | 14 | 22 | 33 | | 47 | 80 |
| Nectar volume | 11 | 14 | 25 | 368 | | 497 | 865 |
| Nectar concentration | 11 | 14 | 25 | 320 | | 433 | 753 |
| Fruit set | 8 | 14 | 22 | 33 | | 47 | 80 |

**Table S2.** VIFs of the remaining variables. Variables have been selected through ‘*stepwise*’ selection based on Variance Inflation Factors (VIF<3) to avoid multicollinearity among the predictor variables.

| **Variables** | **VIF values** |
| --- | --- |
| Branch length | 1.170 |
| Flower diameter | 1.042 |
| Flower length | 1.131 |
| Bract size | 1.007 |
| Bract asymmetry | 1.040 |
| Bract color | 1.080 |

**Table S3**. Detailing of models concerning the differences between morphs in bract and floral traits, including the model parameters and their respective results. The table includes the chi-square test values (χ^2^), *p*-values, estimated coefficients (Est), and standard errors (SE) for each predictor variable tested. Significant *p*-values are expressed in bold. Degrees of freedom = 1 in all cases. Neg. bin. 2: negative binomial distribution. Residual plots are found in figure S2.

|  | Model Parameters | | | Results | | | | |
| --- | --- | --- | --- | --- | --- | --- | --- | --- |
| Nº model | Response variable | Family | Link | Morph | | | | Residuals |
|  | |  | | χ^2^ | p | Est | SE |  |
| M.1 | Bract size | Gamma | Log | 12.21 | **< 0.001** | -0.106 | 0.030 | Fig. S2 - a |
| M.2 | Bract asymmetry | Gamma | Log | 0.053 | 0.817 | -0.001 | 0.005 | Fig. S2 - b |
| M.3 | Bract color | Gamma | Log | 2.574 | 0.108 | 0.159 | 0.099 | Fig. S2 - c |
| M.4 | Flower length | Gamma | Log | 9.938 | **< 0.001** | 0.102 | 0.032 | Fig. S2 - d |
| M.5 | Flower diameter | Gamma | Log | 1.666 | 0.196 | -0.034 | 0.026 | Fig. S2 - e |
| M.6 | Number of flowers | Neg. bin. 2 | Log | 7.806 | **0.005** | -0.217 | 0.077 | Fig. S2 - f |
|  |  |  |  |  |  |  |  |  |

|  | Model Parameters | | | Results | | |  |  |
| --- | --- | --- | --- | --- | --- | --- | --- | --- |
| Nº model | Response variable | Family (link) | Predictor variables | χ^2^ | p | Est | SE | Residuals |
|  |  |  |  |  |  |  |  |  |
| M.7 | Nectar volume (µL) | Gaussian (identity) | Bract size | 12.771 | **< 0.001** | 0.008 | 0.002 | Fig. S2 - g |
|  |  |  | Bract asymmetry | 0.436 | 0.508 | -0.011 | 0.017 |  |
|  |  |  | Flower length | 19.784 | **< 0.001** | 2.299 | 0.517 |  |
|  |  |  | Flower diameter | 0.894 | 0.344 | 2.205 | 2.331 |  |
|  |  |  | Morph | 2.799 | 0.094 | -6.861 | 4.100 |  |
| M.8 | Nectar volume (µL) | Gaussian (identity) | Bract color | 0.005 | 0.938 | -1.932 | 25.191 | Fig. S2 - h |
|  |  |  | Morph | 0.177 | 0.673 | 1.871 | 4.444 |  |
| M.9 | Nectar sugar (mg) | Gaussian (identity) | Bract size | 0.474 | 0.490 | 0.0001 | 0.0001 | Fig. S2 - i |
|  |  |  | Bract asymmetry | 1.793 | 0.180 | -0.001 | 0.0009 |  |
|  |  |  | Flower length | 68.773 | **< 0.001** | 0.168 | 0.019 |  |
|  |  |  | Flower diameter | 5.136 | **0.023** | 0.221 | 0.092 |  |
|  |  |  | Morph | 4.390 | **0.036** | -0.388 | 0.186 |  |
| M.10 | Nectar sugar (mg) | Gaussian (identity) | Bract color | 0.014 | 0.903 | -0.113 | 0.929 | Fig. S2 - j |
|  |  |  | Morph | 0.298 | 0.584 | 0.088 | 0.162 |  |

**Table S4**. Detailing of models concerning the effects of morphological traits on floral resource availability (nectar volume), and nectar quality (nectar sugar), including the model parameters and their respective results. The table includes the chi-square test values (χ^2^), *p*-values, estimated coefficients (Estimate), and standard errors (SE) for each predictor variable tested. Significant *p*-values are expressed in bold. Degrees of freedom = 1 in all cases. Residual plots are found in figure S2.

**Table S5**. Detailing of models concerning the effects of bract traits and resouce on reproductive success, and the effect of bract size on the number of flowers and bract mortality, including the model parameters and their respective results. The table includes the chi-square test values (χ^2^), *p*-values, estimated coefficients (Estimate), and standard errors (SE) for each predictor variable tested. Significant *p*-values are expressed in bold. Degrees of freedom = 1 in all cases. Residual plots are found in figure S2.

|  | Model Parameters | | | Results | | | |  |
| --- | --- | --- | --- | --- | --- | --- | --- | --- |
| Nº model | Response variable | Family (link) | Predictor variables | χ^2^ | p | Est | SE | Residuals |
|  |  |  |  |  |  |  |  |  |
| M.11 | Fruit-set | Binomial (logit) | Bract size | 8.281 | **0.004** | -0.001 | 0.0003 | Fig. S2 - k |
|  |  |  | Bract asymmetry | 2.658 | 0.103 | 0.004 | 0.002 |  |
|  |  |  | Bract color | 1.073 | 0.300 | -2.229 | 2.151 |  |
|  |  |  | Morph | 2.419 | 0.119 | 0.395 | 0.254 |  |
| M.12 | Fruit-set | Binomial (logit) | Nectar volume (µL) | 0.350 | 0.553 | -0.027 | 0.046 | Fig. S2 - l |
|  |  |  | Nectar sugar (mg) | 1.436 | 0.230 | 0.245 | 0.205 |  |
|  |  |  | Morph | 0.014 | 0.903 | 0.085 | 0.706 |  |
| M.13 | Nº Flowers |  | Bract size | 29.405 | **< 0.001** | 0.0006 | 0.0001 | Fig. S2 - m |
| M.14 | Bract mortallity | Binomial (logit) | Bract size | 7.046 | **0.007** | 1.483 | 0.558 | Fig. S2 - n |
|  |  |  |  |  |  |  |  |  |

**Figure S1.** Pairwise correlation coefficients among the predictor variables (bract size, shape, asymmetry, color, flower length, and diameter). The heatmap scale ranges from -1 (strong negative correlation) to 1 (strong positive correlation), with corresponding *p*-values included within each square.


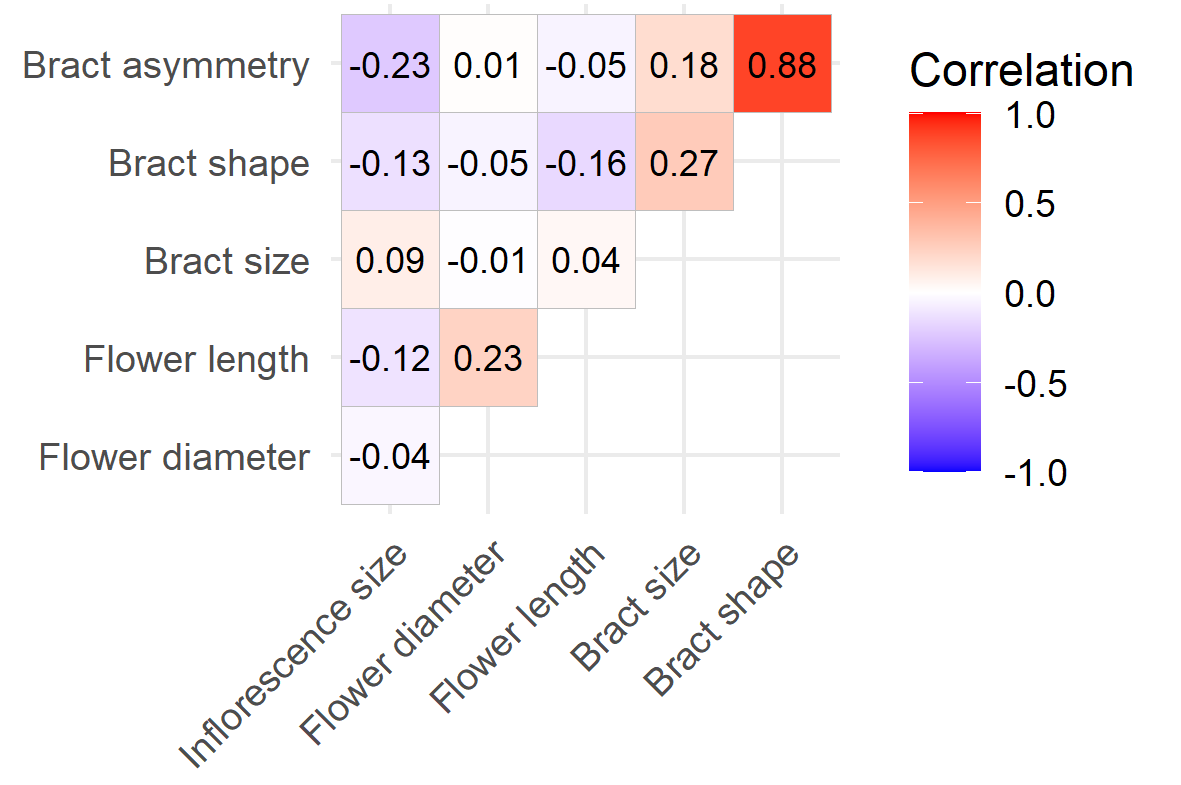


**Figure S2**. Quantile-quantile (QQ) plots of residuals from the models, generated using the *DHARMa* package in R Software. The Kolmogorov–Smirnov test (KS test) evaluates deviation from the expected distribution; the dispersion test checks for overdispersion or underdispersion; and the outlier test detects potential influential observations. Non-significant *p*-values across all tests indicate that residuals are adequately distributed.

(a) Model 1 – GLMM testing bract size differences between floral morphs.


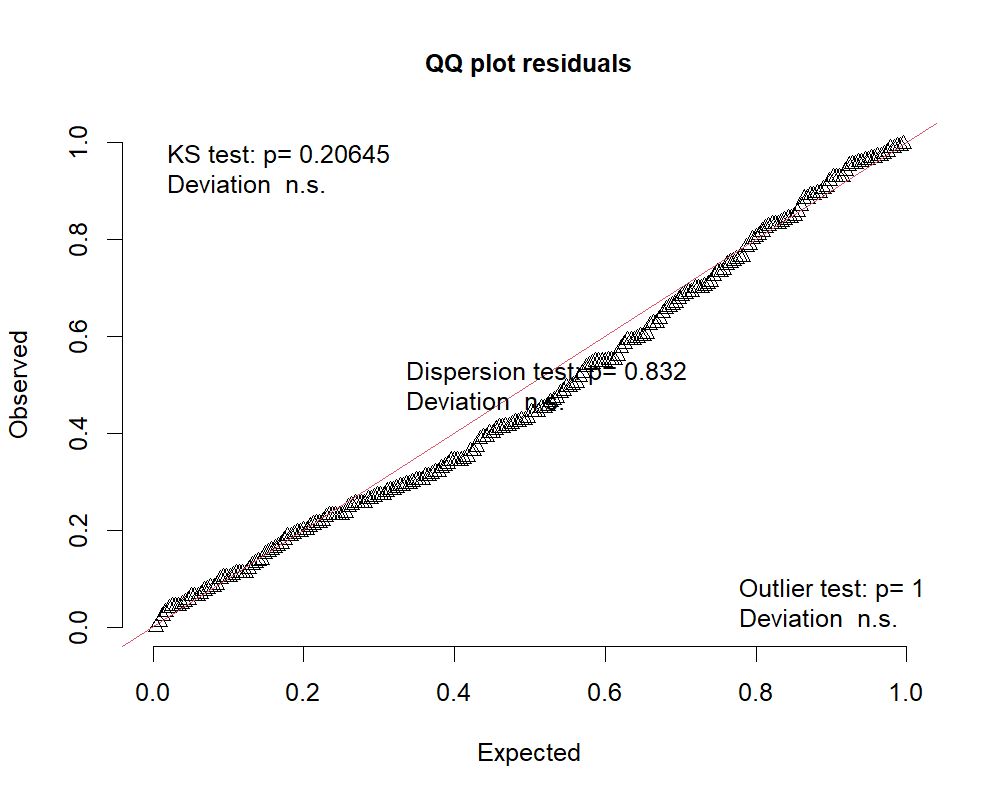


(b) Model 2 – GLMM testing bract asymmetry differences between floral morphs.


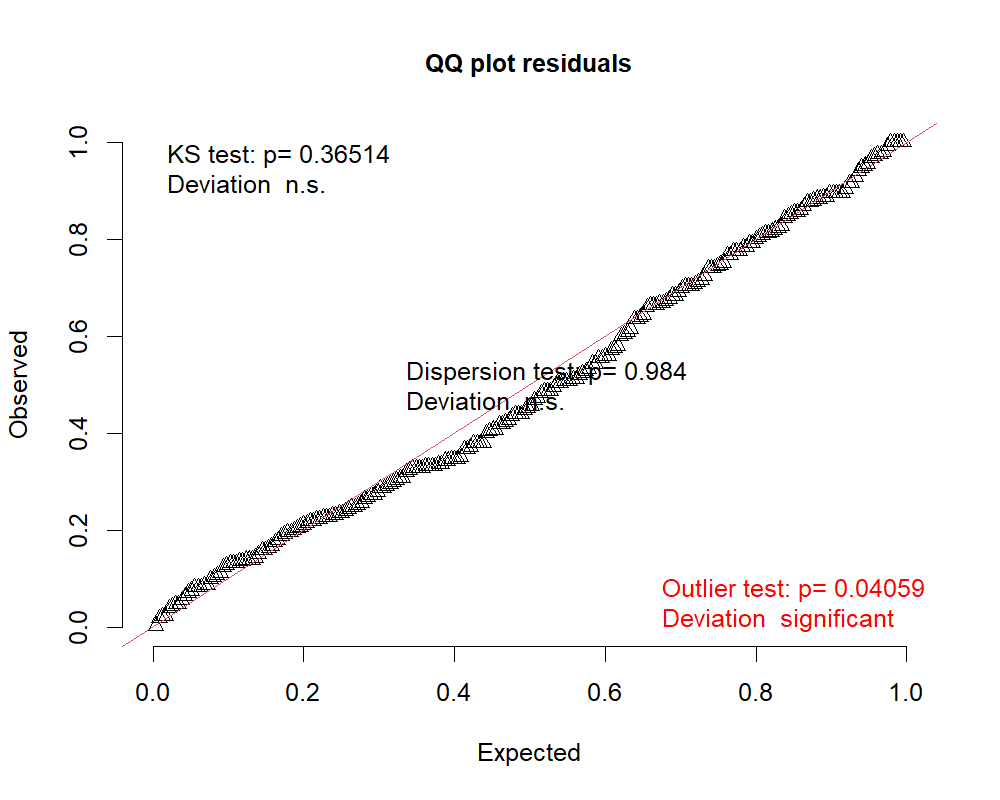


(c) Model 3 – GLMM testing bract color differences between floral morphs.


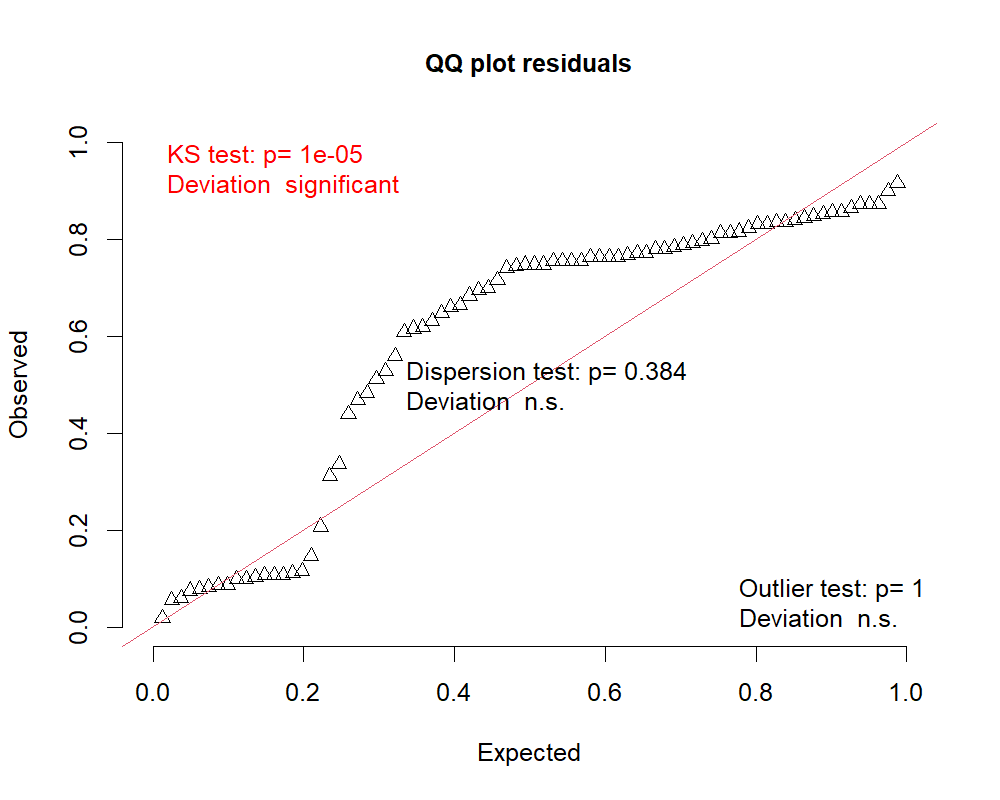


(d) Model 4 – GLMM testing flower length differences between floral morphs.


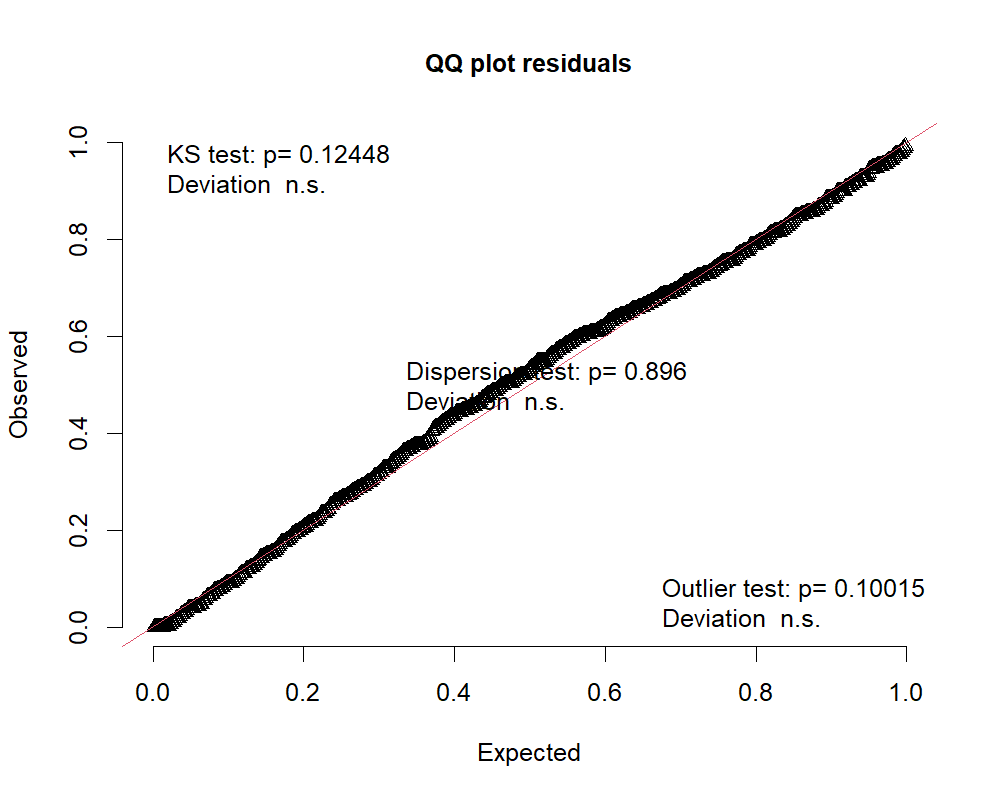


(e) Model 5 – GLMM testing flower diameter differences between floral morphs.


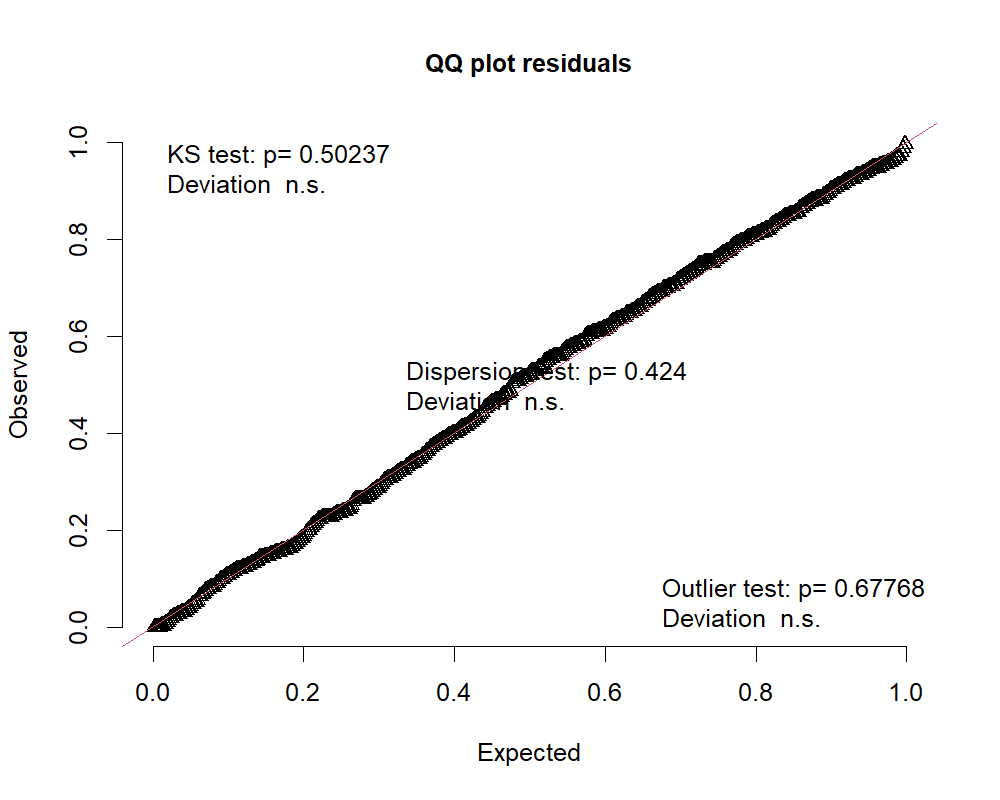


(f) Model 6 – GLMM testing number of flowers differences between floral morphs.


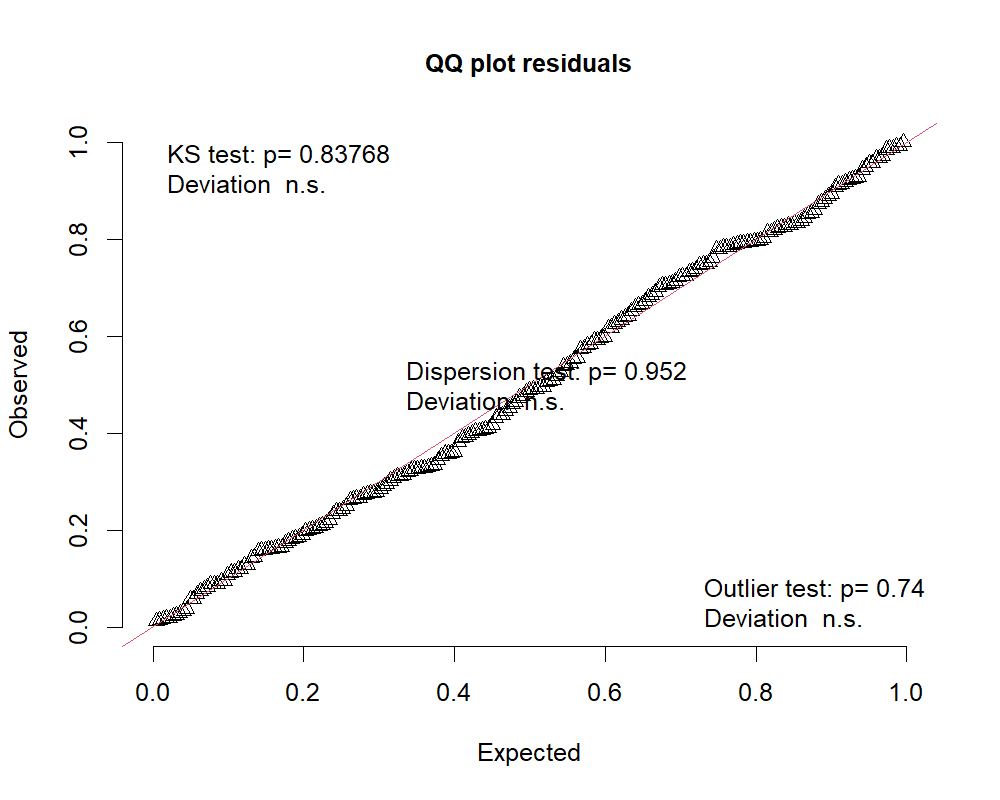


(g) Model 7 – GLMM testing attractive morphological traits related to floral resource availability
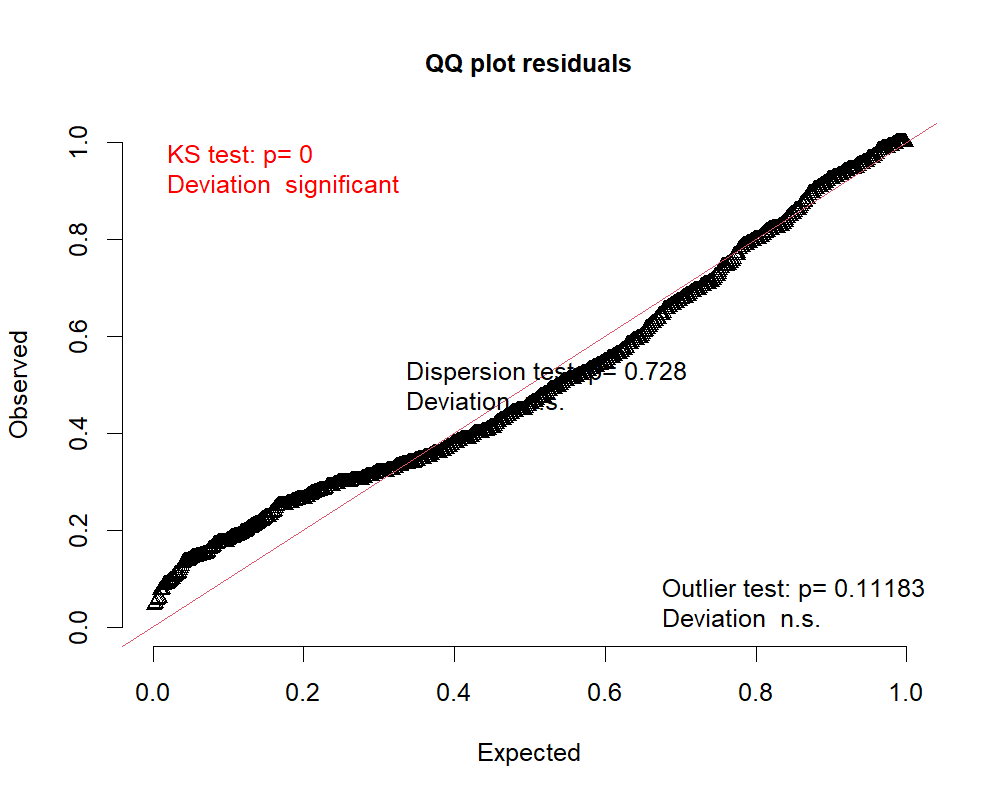
 (nectar volume).

(h) Model 8 – GLMM testing bract color trait related to floral resource availability (nectar volume).


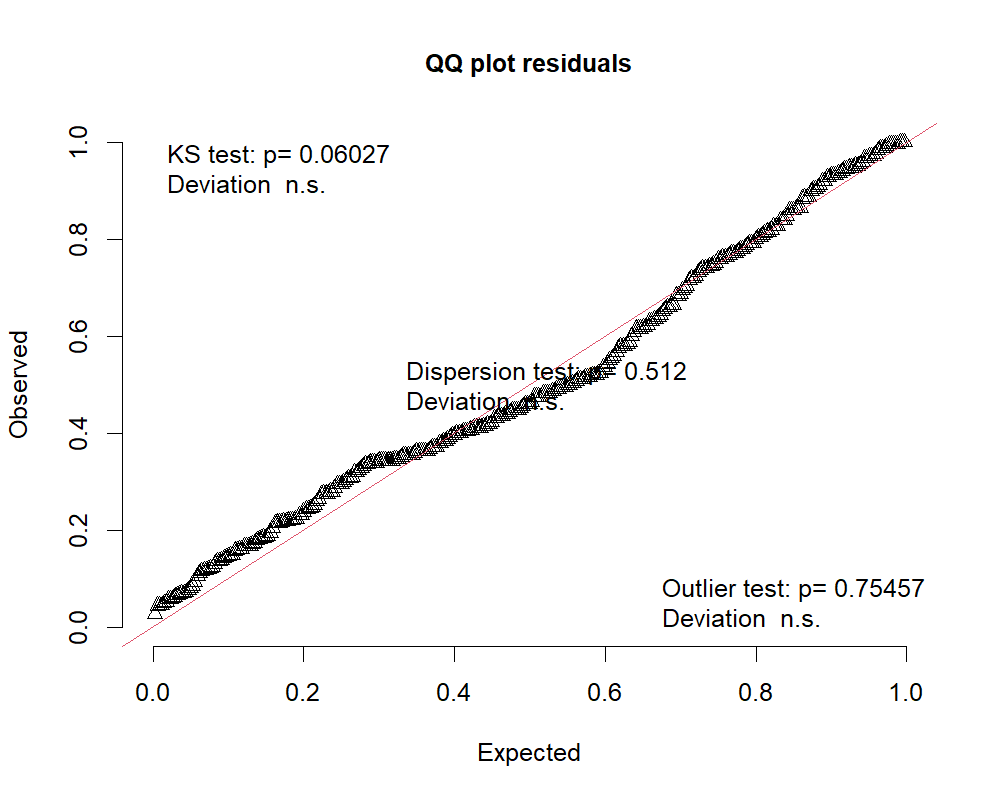


(i) Model 9 – GLMM testing attractive morphological traits related to floral resource quality (nectar sugar).


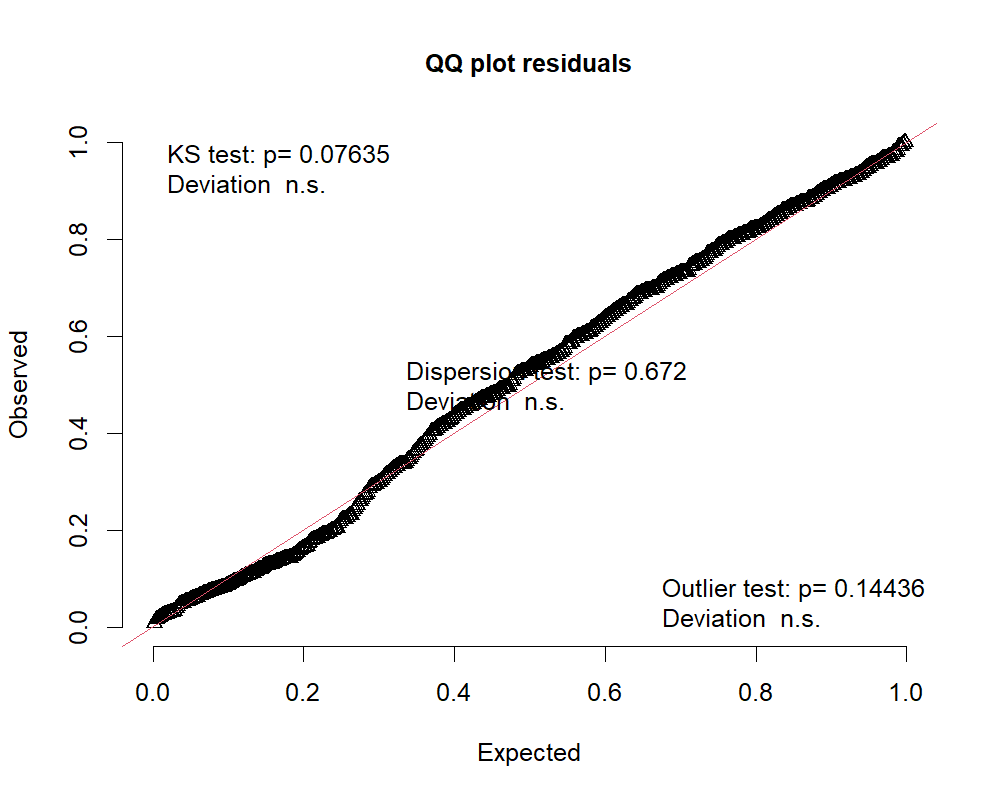


(j) Model 10 – GLMM testing bract color trait related to floral resource quality.


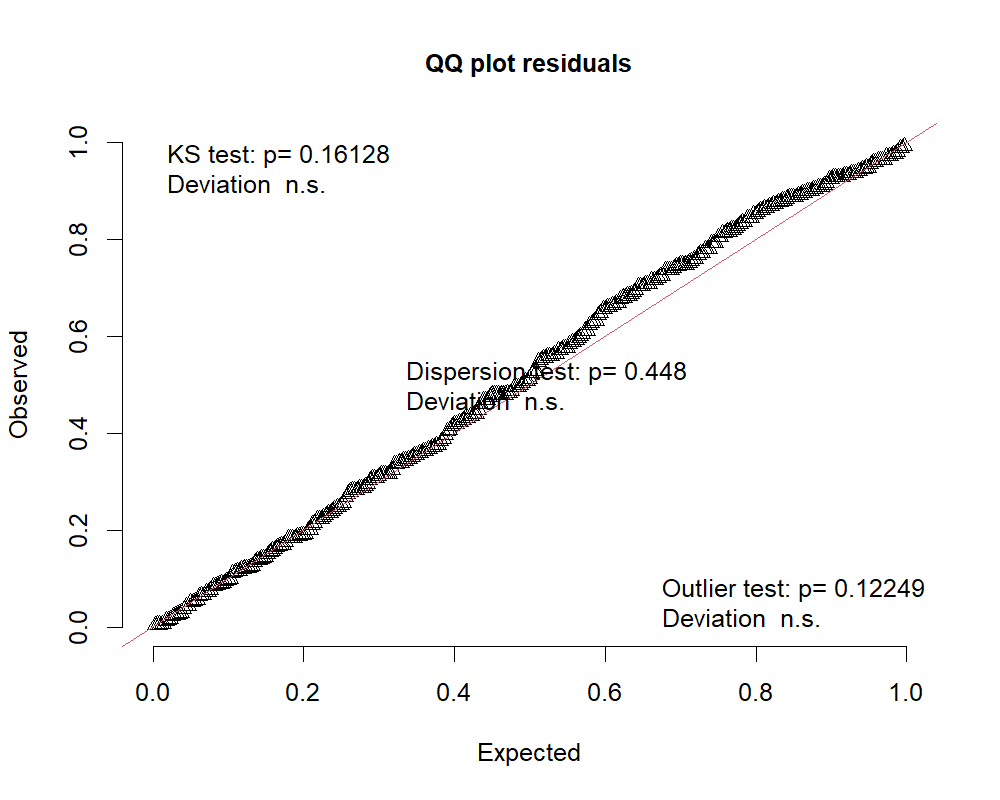


(k) Model 11 – GLMM testing bract traits on reproductive success.


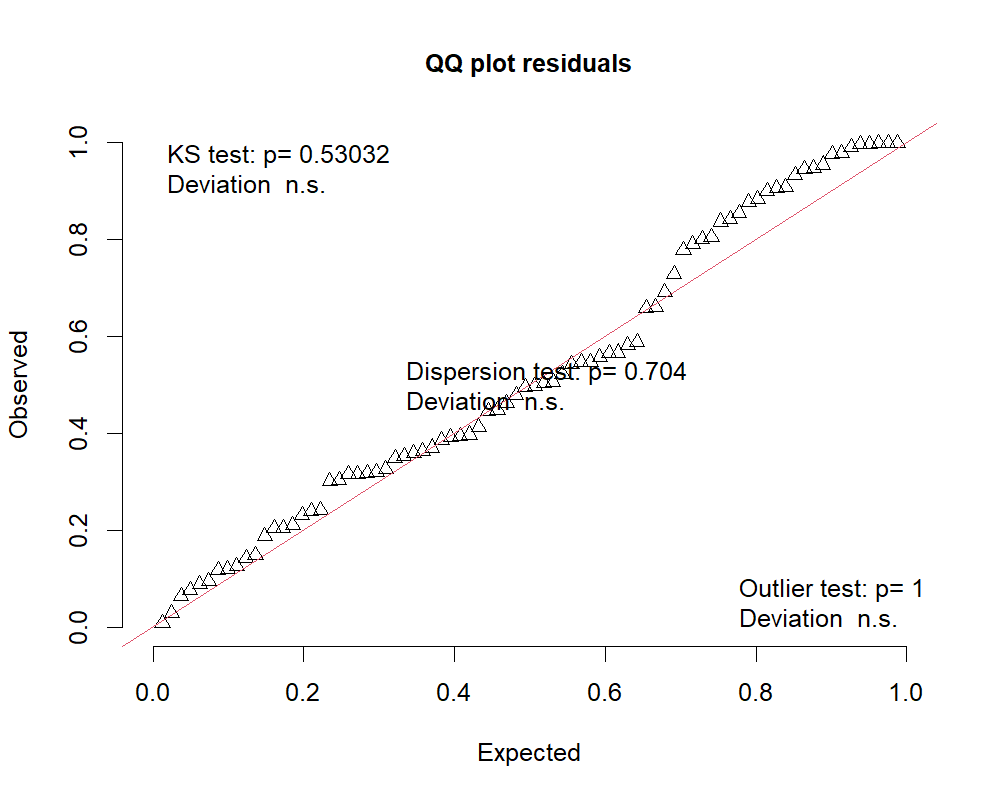


(l) Model 12 – GLMM testing the effect of the nectar availability (quantity) and nectar quality per bract on fruit-set.


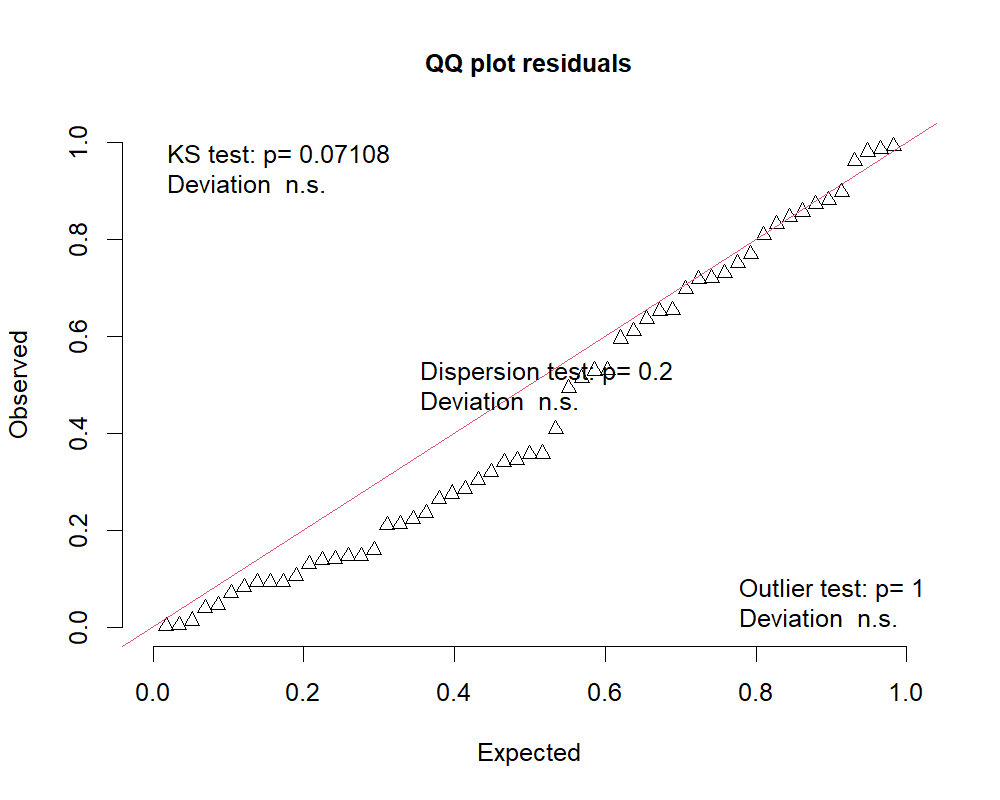


(m) Model 13 – GLMM testing the effect of bract size on number of flowers.


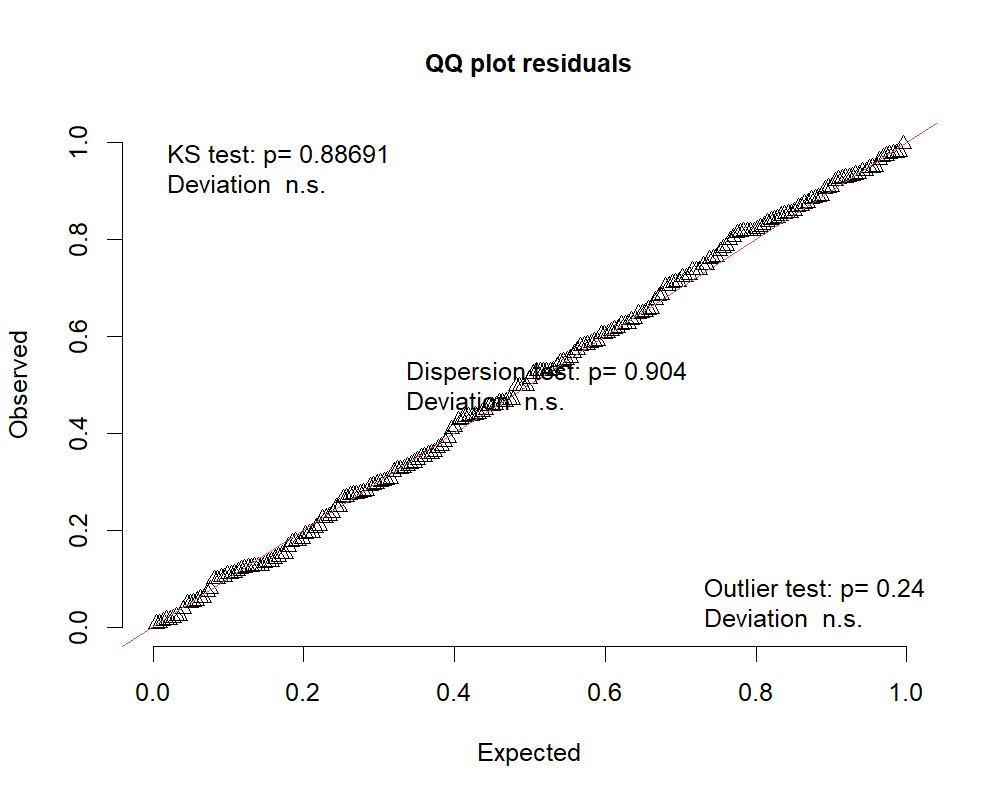


(n) Model 14 – GLMM testing the effect of bract size on bract mortality.


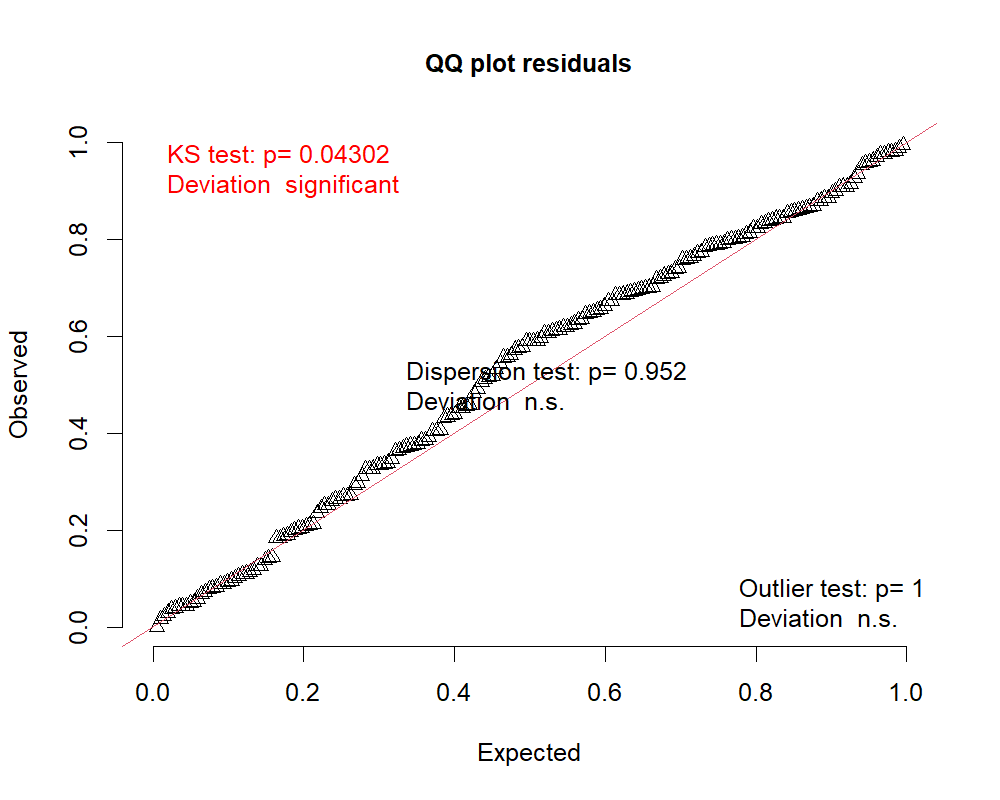

Supplement: Supplementary file 1 — Table S1. Number of plant individuals sampled, and total sample sizes of response variables used on models. Table S2. VIFs of the remaining variables. Variables have been selected through ‘stepwise’ selection based on Variance Inflation Factors (VIF <3) to avoid multicollinearity among the predictor variables. Table S3. Details of models concerning the differences between morphs in bract and floral traits, including the model parameters and their respective results. Table S4. Details of models concerning the effects of attractive morphological traits on floral resource availability (nectar volume), and nectar quality (nectar sugar), including the model parameters and their respective results. Table S5. Details of models concerning the effects of bract traits and resource on reproductive success, and the effect of bract size on the numbers of flowers and bract mortality, including the model parameters and their respective results. Figure S1. Pairwise correlation coefficients among the continuous predictor variables (bract size, shape, asymmetry, colour, flower length, and diameter). Figure S2. Quantile‐quantile (QQ) plots of residuals from the models. [file PLB-27-1478-s001.docx]
